# Too Good to be Nice: The interplay between the Cause marketing and information acquisition

**DOI:** 10.1371/journal.pone.0299157

**Published:** 2024-05-23

**Authors:** Xin Li, Dongsheng Yang

**Affiliations:** 1 Business School, Northwest Normal University, Lanzhou, P.R. China; 2 School of Economics and Management, Hubei University of Arts and Science, Xiangyang, P. R. China; Westminster International University in Tashkent, UZBEKISTAN

## Abstract

Cause marketing (CM) has become an important tool for firms to fulfill their social responsibility strategies. However, in reality, although some consumers have responded positively to the firm’s CM strategies, others have doubts due to their lack of trust in the effectiveness of the firm or business. Therefore, in this paper, we consider a case that the supplier is a dual-purpose corporation that engage in a CM campaign and the consumer social preference is uncertainty and is unknown initially but can be resolved by the retailer’s acquisition behaviour. By examining the two information acquisition strategies: committed acquisition and contingent acquisition. We find that, under either strategy, the retailer would like to acquire information only when the cost of information acquisition is small. Moreover, compared to contingent acquisition, the retailer is more willing to prefer committed acquisition. Additionally, we show that the supplier always prefers the committed acquisition strategy. However, the retailer’s preference toward these two information acquisition strategies is related to the acquire cost. Specifically, when acquisition cost is small or large, the retailer is indifferent between these two strategies, when acquisition cost is in an intermediate range, the retailer will shift her strategy from the contingent strategy to the committed strategy. We also use numerical studies to illustrate main results. These findings provide theoretical support and management insights for managers to integrate CM into business transactions.

## 1 Introduction

As new generations (Millennials and Generation Z) enter the labor market and possess higher purchasing power in recent years, social issues become more prominent than ever before [1]. Even the most powerful firms cannot make business decisions arbitrarily without considering the social issues. According to a leading market intelligence agency Mintel, 73% of Americans consider companies’ social cause when they make their purchasing decisions [2]. To gain better survival and development, while societal marketing programs could take many forms [3], in many emerging markets cause marketing (CM) campaigns were often the first type of societal marketing program to be adopted.

CM, which is also known as cause-related marketing, is an alliance between businesses and non-profit causes to address social issues as well as business objective, and is the practice of donating proceeds from product sales to designated charities [4]. Since the debut of CM in 1981 with the American Express campaign for the Statue of Liberty renovation, CM campaigns have flourished, and a host of companies have employed as a marketing tool to achieve broad range of objectives from controlling the bottom-line performance to strengthening the competitive advantage. Currently, CM is widely used for different purposes. For example, Starbucks gave 50 cents from each sale of the Starbucks RED exclusive beverages to the Global Fund to Fight AIDS, Tuberculosis. Honor donated 1 yuan to the foundation to fund the Dream Center classroom. Apple donated 100% of the eligible proceeds from (PRODUCT)RED purchases to the Global Fund’s COVID-19 Response. Patagonia, gives 1% of their sales to support environmental nonprofit organisations funded at the grassroots level in countries and communities.

Meanwhile, with the decline of government support, non-profit organizations are increasingly turning to cooperate with companies to implement CM in hopes of filling the increasing funding gaps. For example, Nature Conservancy has allied with T-Mobile, Amazon and Bank of America to obtain corporate financial support from the widespread use of CM. It has been reported that CM has become the third-largest category for corporate sponsorship, after sports and entertainment [5]. It has been reported that that US firms’ expenditure on CM initiatives amounted $ 2.23 billion in 2019.

For CM to be successful, it is important for consumers to participate in cause-related product purchasing. Because consumer decision making toward sustainable consumption can be influenced by their ethical philosophy [6]. Previous research has identified “warm glow” as the primary driver of CM, however consumers’ reactions to CM is stochastic, i.e., some consumers respond favourably to companies’ social responsibility initiatives, others apprehend because they do not trust the company or the effectiveness of the cause [7]. For example, Yoplait supported the Susan G. Komen breast cancer foundation by offering $ 0.1 for every yogurt sold, and achieved success. In contrast, KFC collaboration with the Susan G. Komen with the “Buckets for the Cure” campaign by offering $ 0.5 for every KFC bucket sold, resulted in a public outcry, a consumer boycott, and a public relations disaster. Consistently, Cone Millenial Cause Study shown that 78% of U.S. consumers would be more likely to buy a product linked with a social cause and 66% would switch brands to support a cause. In contrast, 51.4% of UK consumers would not willing to pay more for product with ethical attributes and around 22.1% would wish to pay less. Thus, how to effectively identify consumer responses (hereafter called consumer’s social preference as in [8]) to CM is crucial for a firm engaged in CM campaign.

Thanks to the advance of data collection technologies, the company today can choose from a range of methods (such as consumer feedback reports, sales data collection) to access the information about consumers’ social preferences for CM [9]. In practice, the upstream supplier often has less information about consumers’ information than the retailer, because downstream retailers serve consumers directly. As a result, a feasible way for supplier is to rely on the retailer superior market information to make corresponding CM decisions. However, due to the failure of CM campaign, sometimes the downstream retailer is unwilling to promise that he would obtain consumers’ social preference information at the initial stage because information acquisition is time-consuming and costly, such as for purchasing data transmission equipment and processing the collected information. Therefore, the downstream retailer often tends to decide whether or not to acquire consumers’ social preference information after observing the supplier’s CM decision, which may lead to two decision sequences: *contingent acquisition* and *committed acquisition*.

Under the committed acquisition scenario, the retailer first commits to the supplier whether or not to acquire the consumer’s social preference information. Given the retailer’s commitment, the supplier decides the donation amount for each unit sold in the CM campaign. After the product is put in the market, the demand information is realized, and the supplier and the retailer sequentially determine the wholesale price and the retail price. However, if the retailer does not initially adopt the acquisition, the donation and price decisions are made according to the demand expectation. Differently, under the contingent acquisition scenario, the supplier first decision unit donation amount in a CM, and then the retailer decides whether to adopt the acquisition depending on the supplier’s donation level. The pricing stage is similar to that under the committed acquisition scenario, in which both firms can make more precise pricing decisions only if the retailer adopts the acquisition in the prior stage.

The above discussion sheds light on the significance of two strategic decisions, i.e., CM decision and consumers’ social preference information acquisition. However, in a decentralized supply chain, the difference in decision timing between upstream supplier and downstream retailer has an important influence on their profits [10]. Although some papers in operations management have explored the effect of decision timing, the case of combining consumer’s social preference information with suppliers’ CM decision has not yet been addressed in the literature. However, in practice, information acquisition and CM have been widely applied. For example, Alibaba encourages sellers or consumers to participate in charitable donations by setting up different charity projects. At the same time, Alibaba also set its IoT(Internet of Things) platform to collect, analyse and share the market information. Other examples include VF corporation.

Therefore, under the diversity of acquisition timing, this paper investigates the interaction between the supplier’s CM investment and the retailer’s acquisition strategy, in which the supplier is a dual purpose corporation that pursues his own profit as well as social benefit (i.e., the total donation amount to the charity) and the consumer social preference is unknown initially but can be resolved by the retailer’s acquisition behaviour. Based on these assumptions, we try to answer the following questions: (i) How will the retailer’s information acquisition strategy affect the supplier’s CM decision? (ii) Can the retailer induce the supplier to implement CM through information acquisition under different acquisition scenario? (iii) What are the firms’ preferences toward different strategies?

Our research has generated the following interesting findings. First, compared with no information acquisition case, the dual purpose supplier’s always set a higher unit donation amount to charity under both acquisition cases. Moreover, when the retailer’s information acquisition cost is moderate, the dual purpose supplier may set a higher unit donation amount to incentives the retailer to acquire information under the contingent acquisition strategy. Second, when the information acquisition cost is relatively low, the retailer will acquire consumers’ social preference information under either acquisition strategy. Additionally, compared with the contingent acquisition case, the retailer has a stronger motivation to collect consumers’ social preference information under the committed strategy. Third, the supplier always prefers the committed acquisition strategy. However, the equilibrium strategy for the retailer is depending on certain conditions. Specifically, when the cost of information acquisition is either sufficiently low or high, the retailer is indifferent between the two acquisition strategies; otherwise, when the cost of information acquisition is moderate, the retailer will shift her strategy from contingent acquisition to committed acquisition as the acquisition cost increases.

The main contributions of our work are as follows. First, we take a step further to develop a novel quantitative model by incorporating consumers’ social preference uncertainty and asymmetry, which reflects the real practices. As far as I know, this paper is the first to incorporate consumers social preference information asymmetry into the CM design problem. Second, we innovatively investigates the interplay between supplier’s CM and retailer’s information acquisition decisions in a social responsibility supply chain, which is also the first to incorporate information acquisition decisions and their effect into the CM design problem. Third, we shed light on how two acquisition strategies affect the firm donation value and the optimal decisions. Fourth, by contrasting the supply chain members’ utilities under two different acquisition strategies, we put forward some counter-intuitive and novel insights that offer theoretical guidance for companies in the complex environment to formulate efficient CM and offer practical reference significance for government departments to encourage firm engage in CM.

This paper is organized as follows. Section 2 reviews the related literature. Section 3 describes the model. The equilibrium results and acquisition strategies are given in Sections 4 and 5, respectively. Finally, conclusions and future research directions are reported in Section 6. All proofs are presented in the S1 Appendix.

## 2 Literature

The literature related to our work can be categorised into three streams: CM design, dual purpose corporations and information sharing. We now briefly review the related literature in the three areas.

Our work is closely related to the literature on CM design, including pricing [8, 11–13], supply chain coordination [14, 15], information asymmetry [16, 17]) and market entry of socially responsible enterprise [1]. Krishna and Rajan [11] consider spillover effect onto other products in the portfolio to study cause-linked product pricing and show that CM is often associated with price increases to make up for the donation cost. Mallucci et al., [18] examine the effect of reputation concerns on pricing and donation amount in the context of monopoly and duopoly scenarios. The result shows that the effect of reputation concerns on firms’ profit can change dramatically depending on the competitive structure of the market. Li et al., [12] investigate the impact of customer’s reference behaviour on the pricing problem of a cause-linked product. They show that it may be optimal for the firm to decrease the price after the implementation of CM despite the donation cost. Under the setting of asymmetric product quality information, Li and Mallucci [16] examine the informational role of CSR and answer the question of why the high-quality firms sometimes under-invest in CSR compared to lower quality firms. Xu and Li [17] investigate the interplay between e-tailer demand information sharing and supplier cause marketing and show that CM may be a driving factor which motivates the e-tailer to share demand information with supplier under certain conditions.

It is worth mentioning that above aforementioned papers primarily assume that the consumers are homogeneity for CM. In contrast, Gao [8] incorporates consumer preference heterogeneity in both the product itself and the social cause into a game-theoretical model to study firm’s pricing, design and distribution of cause-linked products. But he ignores the possibility of information asymmetry. In fact, in real-life the supply chain often face information asymmetry challenge [19]. To fill this gap, in this paper, we incorporate the consumer social preference asymmetry into a stylised game-theoretical model to explore it effect and the strategic interactions between the supplier’s CM and retailer’s information acquisition strategies.

Our study also relates to the emerging literature on dual purpose corporations. As consumers pay more conscious about social issues, the “beyond profit” managerial objective is gaining momentum. Several empirical papers show that the firm’s responsible concern can serve as a way to differentiate from competitors and increase profitability by attracting more and better customers, employees, and investors [20–22]. For example, Lim [20] study the current situation of consumption research and present some ways to encourage consumers to embrace their inner eco-warrior spirit, fostering sustainable behavior. Fernández and Santaló [21] show that firm’s responsible concern as a device used by firms to differentiate their product from the rival’s products, which is a more valuable strategy in a more competitive market. Some papers also study the economic effect of firm’s responsible concern by building models. For example, Arya et al., [23] examine the effect of dual purpose concern on disclosure incentives. Wang and Li [24] study the effect of downstream firm’s responsible concern on upstream firm’s entry decision. Chen et al., [25] examine the effect of firms’ responsible concern on capacity sharing in duopoly model. Nie et al., [26] investigate the effect of social concern and environmental concern on equilibrium quantity and performance. It is worth noting that the above papers either focus on situations where being socially responsible goes against a company’s self-interest, or where the dual purpose are reflected in its own-self profit plus consumer surplus. In contrast, in this paper, we study CM that is a company’s promotional campaign with the dual purpose of increasing profitability while bettering society. In this sense, our paper is related to the work of Arya and Mittendorf [27]. They consider the case where the firm sets aside some of its output for the social good, and the total donation value is not directly related to the sales volume of the focal product. However, our paper focuses on CM in which the customer purchase triggers the donation, i.e., the total donation value is positively related to the product sales, and highlights the interaction between the supplier’s CM and retailer’s information acquisition decision about consumer’s social preference.

Another stream of related literature is on information acquisition in a supply chain setting. It is well known that supply chain typically contains various forms of information asymmetry (e.g., cost, demand, quality, risk preference, yield, capacity), which drive the nature of business transactions. Therefore, the acquisition of information is undoubtedly very critical in supply chain management. At present, some papers attempt to identify different strategic effects of acquisition. For example, Guo [28] considers the strategic effect of acquisition observability that can influence an upstream seller’s pricing behaviour by means of voluntary information disclosure. Guo and Iyer [29] explore a sequential acquisition problem and show that if the manufacturer can continuously acquire the market information and disclose it to the downstream retailer, he may choose not to acquire the information even if it is costless to do so. Guan and Chen [30] investigate the impact of acquisition on the retailer’s quality speculation process and show that the manufacture may give up any consumer information, even when such acquisition is costless. Xiao et al., [31] consider the interaction between the retailer’s information acquisition strategies and the manufacturer’s quality enhancement decision. Wu et al., [32] extend Xiao et al.,’s [31] work to an E-commerce case and investigate the interplay between the supplier’s quality enhancement and the online retailer’s quality preference information acquisition decisions under a dual-channel setting.

Our work follows the these aforementioned papers but differs in several respects. First, our study focuses on the emerging business environment in which firms not only focus on their own interests but also the interests of other stakeholders, and our aim is to investigate the interaction between the dual-purpose supplier’s CM and for-profit retailer’s information acquisition of consumers’ social preference. Second, we shed light on the timing issue between two common acquisition strategies in practice and examine the retailer’s information acquisition conditions and the firms’ preferences between two acquisition strategies. Third, our study derives several interesting results as well as new management insights that are absent in the existing CM literature. We believe that our study can complement the literature regarding the design of CM or CSR and can provide both marketers and firm managers with a better understanding and guidance on CM implementation. In summary, the distinction between the our paper and several related studies is outlined in Table 1.

**Table 1:** The paper in comparison with the main literature.

Paper	Structure	Information		Research topic
		Asymmetry	Category	
Heydari and Mosanna [14]	1S1R	/	/	supply chain coordination
Modak et al., [33]	1S1R	/	/	supply chain coordination
Gao [8]	1S,1S1R	/	/	pricing and design of CM product
Korpeoglu et al., [1]	2R	/	/	entry of socially responsible retailer
Li and Mallucci [16]	1S	✓	quality	supply chain coordination
Xu and Li [17]	1S1R	✓	demand	the interplay between CM and IS
Mosanna et al., [7]	1S1R	✓	social awareness	Supply chain coordination
Wu et al., [38]	1S1R	/	/	Selection of CM
This paper	1S1R	✓	social preference	information acquired strategy

S: supplier; R: retailer; IS: information sharing.

## 3 The model

Consider a standard supply chain where a supplier (she) sells the product through a for-profit retailer (he). In addition to choosing the wholesale price, the supplier also decides donation amount for each unit sold in a CM campaign to maximize a linear combination of own profit and social benefit (after called dual-purpose supplier). Similar to [24], we normalize the weight the supplier places on profit to 1, while denote by *θ* the weight the supplier places on charity, where *θ* ∈ [0, 1). The objective function (utility function) for the dual-purpose supplier and the for-profit retailer are

$$\begin{array}{c} v_{s}=\pi_{s}+\theta \Lambda ,\;\;\;v_{r}=\pi_{r}, \end{array}$$ (1)

where *π*_*s*_(*π*_*r*_) is the profit for the supplier (retailer), and Λ is the total donation amount, which is equal to the multiplication of the unit donation amount *k* and the demand *d*, i.e., Λ = *kd*. Note that the objective function for the dual-purpose is the profit plus the total donation amount rather than the profit plus consumer surplus in this paper, this is because the CM is a company’s promotional campaign with the dual purpose of increasing profitability while bettering society (such as Non-profit Organization), not just consumers. A larger *θ* represents the scenario where the supplier cares more about social benefit, whereas the special case of *θ* = 0 corresponds to the scenario of a purely for-profit supplier studied in the literature, such as [8, 17]. Without loss of generality, as in [16, 17], we normalize both the supplier’s production cost and the retailer’s selling cost to zero.

### 3.1 The demand function

In CM, the warm glow is thought to be one of the main drivers of the positive effect on consumers. The present works show that the consumer’s appraisal of the charity increases warm glow per donation dollar [8, 11, 34]. Thus, with reference to [11, 33, 35], when the dual-purpose supplier donates *k* dollars to a designated charitable cause for each unit sold, each consumer experiences an increase in utility *βk* from purchasing the cause-linked product. In fact, we can easy to see that the optimal decisions remains robust when some consumers are prosocial and the rest are non-prosocial [17]. Therefore, consumption utility includes the now-standard elements for cause marketed products, i.e., product value, price and warm glow, which is given by

$$\begin{array}{c} u=v+\beta k-p, \end{array}$$ (2)

where *v* is the valuation of the product and is assumed to be exogenous, *β* ≥ 0 is consumer’s preference for social cause [8], or consumer social awareness level [7]. To capture such positive variations, we follow the approach used by [31, 32] and assume that *β* follows a uniform distribution between 0 and 1: *β* ∼ *U*[0, 1] with probability 1/2, or between 1 and 2: *β* ∼ *U*[1, 2] with probability 1/2. This assumption not only captures the consumer’s preference for the social cause is heterogenous, but also for analytic tractability. Note that this assumption is used to clarify the interaction between the supplier’s CM and retailer’s information acquisition decisions, and to reveal more clearly managerial implications. We further assume that consumers can always learn their social preference initially and buys the product only if their surplus is above zero, i.e., *u* ≥ 0. However, both the supplier and the retailer only hold the same prior belief that the consumer’s social preference belongs to above two possible ranges with equal probability. This implies that:

(a) If the retailer acquires the consumer’s social preference information and finds that the consumer preference level is low, i.e., *β* ∼ *U*[0, 1], the demand function is given by $D_{l}\left(k,p\right)=1-\frac{p-v}{k}$;
(b) If the consumer preference level is high, i.e., *β* ∼ *U*[1, 2], the demand is given by $D_{h}\left(k,p\right)=2-\frac{p-v}{k}$;
(c) If the retailer does not acquire consumer’s social preference information, i.e., *β* ∼ *U*[0, 2], the demand function is given by $D_{n}\left(k,p\right)=1-\frac{p-v}{2k}$.

### 3.2 Information acquisition and timeline of the game

In practice, to understand the consumer preference and resolve the market uncertainty, the retailer often uses various methods (e.g., Conducting a survey in the market, asking people how they like the product and the social cause, and using statistical methods to calculate relevant metrics [8]) to acquire the consumer’s preference information. In general, these acquisition strategies are usually costly, and we define the cost of information acquisition as *c*. Meanwhile, without loss of generality, we assume that when the retailer acquires the costly information, i.e., he is able to confirm the exact range of the consumer’s social preference.

**Fig 1:**
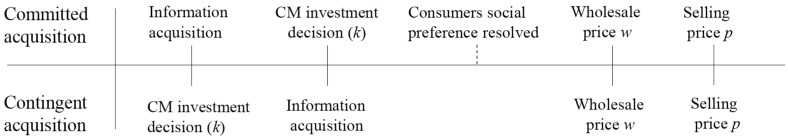
The timeline of game.

Depending on whether the retailer commits to acquiring consumer’s social preference information before or after the supplier sets the donation decision, in this paper we highlights two alternative acquisition strategies: committed acquisition and contingent acquisition, which are widely used by companies to solve problems involving asymmetric information in practice [32]. Specially, under the committed acquisition strategy, the retailer first decides whether to acquire the consumer’s social preference information. Then after observing the retailer’s information acquisition decision, the dual-supplier decides the donation amount *k*, which is also observed by the retailer. Third, the consumer’s social preference is observed by the retailer and shared with the supplier. Fourth, the supplier sets the wholesale price *w*. Finally, the retailer determines the retail price *p*. By contrast, under the contingent acquisition strategy, the supplier first determines the donation level *k*, and then the retailer determines whether to acquire consumer’s social preference information. The rest of the game remains the same as that in the scenario of the committed acquisition strategy. Note that if the retailer chooses disclosure information, then the disclosed information must be truthful. This truthful revelation policy is widely adopted in the literature [36, 37] and can be enforced by the third party verifications or hard evidences. Based on the above discussion, the decision sequence under two different strategies is described in Fig 1.

Since the game contains multiple stages of strategic interactions between the manufacturer and the retailer, backward induction is applied to ensure subgame perfection throughout this paper. Note that under the scenario of either committed acquisition or contingent acquisition, we assume that the supplier’s CM decision is made before pricing decision. This assumption captures CM campaigns, where the firm initially enters into an agreement with selected charity about the terms of the donation. These agreements do not usually include pricing terms, with the firm retaining control of the pricing decision. Further, the prices of donation embedded products change more frequently than do the size of the donation [11, 12].

## 4 Analysis

To understand the effect of consumer homogeneous social preference on CM and how the interactive impact of supplier’s CM and retailer’s information acquisition decisions, in this section we first derive the donation and pricing decisions under the committed acquisition and contingent acquisition scenarios, respectively. Then, by comparing the firms’ expected profits, we present the equilibrium strategy from either firm’s perspective.

### 4.1 Committed acquisition

Under committed acquisition strategy, the for-profit retailer first commits the dual-purpose supplier that he will obtain the consumers’ social preference information before observing the supplier donation decision. Recall that both the donation and consumers’ social preference (if acquired) are resolved before the pricing. Thus, we first assume that the retailer has made his information acquisition decision, and accordingly derive the supplier’s donation amount decision. By comparing his expected payoffs with and without acquisition behaviours, we characterize the retailer’s equilibrium information acquisition condition.

#### Non-acquisition

If the retailer gives up to acquire consumers’ social preference information, then both the supplier and he hold the same prior belief, i.e., the consumers’ social preference may fall into two ranges [0, 1] or [1, 2] with equal probability. We use the superscript *N* to indicate variables for the case of non-acquisition. Given the unit donation amount *k*, the utility functions for the supplier and the retailer are

$$\begin{array}{c} v_{s}^{N}=\left(w-k\right)\left(1-\frac{p-v}{2k}\right)+\theta k\left(1-\frac{p-v}{2k}\right),\;\;v_{r}^{N}=\left(p-w\right)\left(1-\frac{p-v}{2k}\right). \end{array}$$ (3)

According to the standard derivation procedure, the equilibrium wholesale price and retail price can be derived as

$$\begin{array}{c} w\left(k\right)=\frac{v+(3-\theta )k}{2},\;\;p\left(k\right)=\frac{3v+(7-\theta )k}{4}, \end{array}$$

Obviously, the wholesale and retail prices increase monotonously with donation amount *k*, this is because a large donation amount increases the consumers’ willing to pay for the product due to warm glow effect [4], which means ensuring market share, thereby increasing both the wholesale and retail prices. Anticipating the price decisions, the supplier chooses the donation amount *k* to maximize $v_{s}^{N}\left(k,p\left(k\right),w\left(k\right)\right)$, leading to the optimal donation amount

$$\begin{array}{c} k^{N*}=\frac{v}{1+\theta }. \end{array}$$

Underpinned by the above decision making on the equilibrium retail price, wholesale price, and donation amount, the expected utilities of the dual-purpose supplier and for-profit retailer are given by

$$\begin{array}{c} v_{s}^{N*}=\frac{v(1+\theta )}{4},\;\;v_{r}^{N*}=\frac{v(1+\theta )}{8}. \end{array}$$

#### Acquisition

When the retailer promises to acquiring the consumers’ social preference information ex-ante, the supplier know that the consumers’ private social preference level, *β*_*i*_, will be resolved before the pricing stage despite that she still has to set the donation amount based on the expected level of consumer social preference. We use the superscript *A* to indicate variables for the case of acquisition. Therefore, at the pricing stage, given the donation amount *k* and consumers’ social preference level *β*_*i*_, *i* ∈ {*h*, *l*}, the utility functions for the dual-purpose supplier and for-profit retailer are given by

$$\begin{array}{c} v_{s}^{A}\left(k,\beta_{h}\right)=\left(w-k\right)\left(2-\frac{p-v}{k}\right)+\theta k\left(2-\frac{p-v}{k}\right),\;\;v_{r}^{A}\left(k,\beta_{h}\right)=\left(p-w\right)\left(2-\frac{p-v}{k}\right)-c, \end{array}$$

and

$$\begin{array}{c} v_{s}^{A}\left(k,\beta_{l}\right)=\left(w-k\right)\left(1-\frac{p-v}{k}\right)+\theta k\left(1-\frac{p-v}{k}\right),\;\;v_{r}^{A}\left(k,\beta_{l}\right)=\left(p-w\right)\left(1-\frac{p-v}{k}\right)-c. \end{array}$$

Considering a similar procedure as that in the scenario of non-acquisition, when the consumer preference for social cause is high, i.e., *β* = *β*_*h*_, the optimal wholesale price and retail price are

$$\begin{array}{c} w\left(k,\beta_{h}\right)=\frac{v+(3-\theta )k}{2},\;\;p\left(k,\beta_{h}\right)=\frac{3v+(7-\theta )k}{4}. \end{array}$$

In contrast, when the consumer’s social preference level is low, i.e., *β* = *β*_*l*_, the optimal wholesale price and retail price are

$$\begin{array}{c} w\left(k,\beta_{l}\right)=\frac{v+(2-\theta )k}{2},\;\;p\left(k,\beta_{l}\right)=\frac{3v+(4-\theta )k}{4}. \end{array}$$

Given that the supplier is not aware of consumers’ preference information for social cause, she sets the donation amount *k* by combining the above optimal decisions to maximize his expected utility

$$\begin{array}{c} \frac{1}{2}v_{s}^{A}\left(k,w\left(k,\beta_{h}\right),p\left(k,\beta_{h}\right)\right)+\frac{1}{2}v_{s}^{A}\left(k,w\left(k,\beta_{l}\right),p\left(k,\beta_{l}\right)\right). \end{array}$$

Therefore, the optimal donation level can be derived as

$$\begin{array}{c} k^{A*}=\frac{2v}{\sqrt{2+4\theta +4\theta^{2}}}. \end{array}$$

Correspondingly, the equilibrium results for the dual-purpose supplier and for-profit retailer under information acquisition are, respectively,

$$\begin{array}{c} v_{s}^{A*}=\frac{v[1+2\theta +\sqrt{2+4\theta +4\theta^{2}}]}{8},\;\;v_{r}^{A*}=\frac{v[1+2\theta +\sqrt{2+4\theta +4\theta^{2}}]}{16}-c. \end{array}$$

**Proposition 1** Compared with no-information acquisition case, the dual-purpose supplier sets a higher donation level with information acquisition.

Proposition 1 shows that, compared with the case when the retailer does not to acquire consumer’s social preference information, the dual-purpose supplier has an incentive to set a high unit donation amount when the retailer chooses to obtain consumers’ social preference information. This seems intuitive. Interestingly, although the dual-purpose supplier make donation decision before resolving the consumers’ social preference information under both information acquisition strategies, we find that the results are different between these strategies. The reason behind this is that if the retailer promises to obtain information about consumers’ social preferences ex ante, the dual-purpose supplier can confirm the information about consumers’ social preferences before making wholesale price decisions. Even when the consumer’s social preference is low, the supplier can ensure her utility by changing the wholesale price. It is this predictable utility enhancement that will motivate dual-purpose supplier to increase the unit donation level.

Based on the above discussion, we can infer that the dual-purpose supplier’s investment in CM campaign is not driven by highly accurate preference information. Instead, the upstream supplier is more likely to CM merely because she secures the commitment of the retailer to obtain consumers’ social preference information. This finding may be ascribed to the fact that the upstream supplier obtains more accurate consumer social preference information that can push her into setting a proper donation value and subsequently obtaining more net surplus from consumers. These trends will indirectly stimulate the supplier to invest more donation cost to expanding its market potential and obtaining more profit from CM campaign.

**Proposition 2** With committed acquisition, there exist an acquisition cost threshold *c*_1_ such that:

(a) If *c* ≤ *c*_1_, then the retailer acquires information, and the optimal donation level and expected utilities are $k^{*}=\frac{2v}{\sqrt{2+4\theta +4\theta^{2}}}$, $\pi_{s}^{*}=\frac{v[1+2\theta +\sqrt{2+4\theta +4\theta^{2}}]}{8}$ and $\pi_{r}^{*}=\frac{v[1+2\theta +\sqrt{2+4\theta +4\theta^{2}}]}{16}-c$.
(b) If *c* > *c*_1_, the retailer does not acquire information, and the optimal donation level and expected utilities are $k^{*}=\frac{v}{1+\theta }$, $\pi_{s}^{*}=\frac{v(1+\theta )}{4}$ and $\pi_{r}^{*}=\frac{v(1+\theta )}{8}$.

Proposition 2 represents the information acquisition decision of the retailer. The results show that the retailer’s information acquisition strategy exhibits a cut-off structure. Specifically, the retailer commits to acquiring information only if the acquisition cost is below a threshold, above which he commits to not acquiring information. From the perspective of the retailer, the benefits brought by having better access to consumers’ social information via acquisition outweigh the losses brought by the acquisition cost if such cost is relatively low. Therefore, the retailer prefers the acquisition strategy over the non-acquisition strategy under such conditions. Otherwise, the retailer does not commit to obtaining consumers’ preference information for social cause.

### 4.2 Contingent acquisition

Unlike the scenario of committed acquisition, where the retailer has to ex-ante commit to he acquisition decision, under the contingent acquisition scenario, the retailer can make her acquisition decision after observing the supplier’s donation decision. Nevertheless, the dual-purpose supplier has to determine the donation level first in anticipation of the retailer’s acquisition decision. With backward induction, the derivation process of the firms’ equilibrium decisions and acquisition strategies follows a similar principle as the Section 4.1. Here, we directly present the firms’ equilibrium strategies directly in the following proposition.

**Proposition 3** With contingent acquisition, there exist acquisition cost thresholds *c*_2_ and *c*_3_ such that:

(a) If *c* ≤ *c*_2_, the retailer acquires consumers’ social preference information, and the optimal donation level and expected utilities are $k^{*}=\frac{2v}{\sqrt{2+4\theta +4\theta^{2}}}$, $\pi_{r}^{*}=\frac{v[1+2\theta +\sqrt{2+4\theta +4\theta^{2}}]}{16}-c$, $\pi_{s}^{*}=\frac{v[1+2\theta +\sqrt{2+4\theta +4\theta^{2}}]}{8}$;
(b) If *c*_2_ < *c* ≤ *c*_3_, the retailer acquires consumers’ social preference information, and the optimal donation level and expected utilities are $k^{*}=\frac{16c-\theta v-4\sqrt{16c^{2}-2c\theta v}}{\theta^{2}}$, $\pi_{s}^{*}=h\left(\theta \right)$, $\pi_{r}^{*}=\frac{h(\theta )}{2}-c$, where $h\left(\theta \right)=\frac{8\sqrt{16c^{2}-2vc\theta }\left[16\left(2\theta^{2}+2\theta +1\right)c-\theta v\left(1+\theta \right)\right]-512c^{2}\left(2\theta^{2}+2\theta +1\right)-32v\theta c\left(2\theta^{2}+3\theta +2\right)-\theta^{2}v^{2}}{16\theta^{2}\left(16c-\theta v-4\sqrt{16c^{2}-2c\theta v}\right)}$;
(c) If *c* > *c*_3_, the retailer does not acquire consumers’ social preference information, and the optimal donation level and expected utilities are $k^{*}=\frac{v}{1+\theta }$, $\pi_{r}^{*}=\frac{v(1+\theta )}{8}$, $\pi_{s}^{*}=\frac{v(1+\theta )}{4}$.

Proposition 3 presents the firm’s equilibrium strategies under the contingent acquisition strategy. Analogous to the committed strategy, the retailer’s information acquisition strategy exhibits a cut-off structure. When the acquisition cost is low (i.e., *c* ≤ *c*_1_), the retailer is always willing to acquire consumers’ social preference information. This conclusion is expected. On the one hand, the retailer can make more effective decisions at the pricing stage. On the other hand, the supplier can set the same level of equilibrium donation amount as that under the committed strategy. Therefore, when the cost of information acquisition is small, he opts to obtain consumers’ social preference information. When the acquisition cost reaches a moderately range (i.e., *c*_1_ < *c* ≤ *c*_3_), the retailer’s willingness of choosing information acquisition weakens. Under such a circumstance, the supplier has no choice but to raise the donation amount to provide stronger incentives for the retailer to acquire information. Thus, we observe an interesting phenomenon that the donation amount is increasing with the acquisition cost *c*. This can be interpreted as the dual-purpose supplier having to partially cover the retailer’s acquisition cost via consumers’ warm glow effect. Finally, when the acquisition cost is sufficiently high (i.e., *c* > *c*_3_), it is no longer profitable for the dual-purpose supplier to raise the donation level to incentive the retailer. As a consequence, the supplier would choose a low donation amount to save the donation cost, and the retailer chooses not to acquisition accordingly.

In comparison with Proposition 1, we find a significant difference in Proposition 3, i.e., when the cost of information acquisition lies in a relatively moderate range, the retailer chooses to obtain consumers’ social preference information only when the supplier invests enough in CM campaign. One can interpret the inherent difference between these two acquisition timing scenarios: the credibility of retailer’s commitment on acquisition. That is, when the acquisition cost is either low or high, the retailer is able to credibly commit to information acquisition or non-acquisition, which makes no difference between the two scenarios. However, when the acquisition cost is moderate, the retailer cannot credibly commit to social preference information acquisition unless the dual-purpose supplier can invest a sufficiently high donation amount to induce the retailer to acquire information.

## 5 Comparison

In the above section, our study obtains the dual-purpose supplier’s equilibrium decision and the retailer’s information acquisition decision under the committed and contingent strategies, respectively. In this section, we further investigate how the change in acquisition timing can influence the retailer’s acquisition incentive, the supplier’s donation level, and the firm’s utilities.

**Proposition 4** Comparing the equilibrium decisions under committed acquisition and contingent acquisition strategies,

(a) the retailer has a stronger incentive to acquire information under committed acquisition than that under contingent acquisition;
(b) the optimal donation level is higher under contingent acquisition when *c* ∈ (*c*_1_, *c*_3_), and the optimal donation level is higher under committed acquisition when *c* ∈ (*c*_2_, *c*_1_).

Proposition 4 (a) presents the retailer’s information acquisition incentive under the two strategies. Recall that Proposition 2, the retailer prefers to obtain consumers’ social preference information only if *c* < *c*_1_. Moreover, under the contingent strategy, the retailer opts to obtain consumers’ social preference information only if *c* < *c*_3_ in accordance with Proposition 3. Obviously, *c*_3_ is always higher than *c*_1_ due to the strategic influence of decision timing on retailers’ acquisition behaviour. Specifically, under the committed acquisition strategy, the retailer anticipates that the acquisition of consumers’ social preference information will not only enhance he decision making at the pricing stage but also stimulate the dual-purpose supplier to increase CM invest, which in turn further motivates the retailer to obtain preference information of social issue. Therefore, the retailer initially opts to decide on the information acquisition. By contrast, under the contingent acquisition strategy, the motivation of the retailers’ information acquisition depends on the investment of the dual-purpose supplier in CM campaign. A higher donation amount corresponds to a stronger motivation for the retailer to obtain consumers’ preference information. However, when the information acquisition cost reaches a certain level, the dual-purpose supplier gives up her CM invest optimization, and the retailer refuses to obtain consumers’ social preference information accordingly.

Proposition 4 (b) compares the equilibrium donation level under the two strategies. Results show that when consumers’ social preference acquisition cost is intermediate (i.e., *c*_1_ < *c* < *c*_3_), the donation level under contingent acquisition is higher than that under committed acquisition, because in this range, the dual-purpose supplier has to invest more in CM campaign to incentives the retailer to obtain consumers’ social preference information. Therefore, the donation level is increasing in acquisition cost and is stronger under contingent acquisition than under committed acquisition. By contrast, under the committed strategy, the donation level under information acquisition is higher than that under no-acquisition in accordance with Proposition 1, but this level has nothing to do with acquisition cost. When the acquisition cost pushes the retailer into obtaining consumers’ social preference information under the committed strategy and refusing to obtain such information under the contingent acquisition strategy (i.e., *c*_2_ < *c* < *c*_1_), the optimal donation level under the committed strategy is larger than that under the contingent strategy.

**Fig 2:**
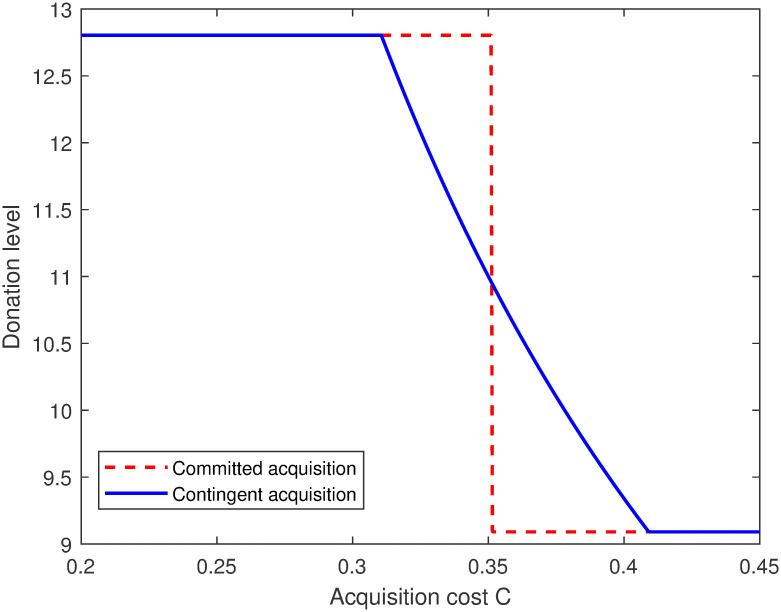
The optimal donation level.

With reference to [38], a numerical example is presented to show managerial insights of Proposition 4 as shown in Fig 2. We employ numerical studies with basic parameters: *v* = 20, *θ* = 0.1 (these parameters are used through out the paper if no otherwise specified). We also tried a wide variety of parameter values for this plot, the results follow analogous qualitative patterns.

From the Fig 2, we find that the optimal donation level can be higher under the committed acquisition strategy when the acquisition cost falls into the intermediate range (e.g., *c* ∈ (0.32, 0.35). In this range, the supplier induces the retailer to acquire the consumers’ social preference information. In contrast, when the acquisition cost is large (e.g., *c* ∈ (0.35, 0.41)), the change in decision timing makes the retailer adopt acquisition under the contingent acquisition scenario but adopt non-acquisition under the committed scenario. Thus, the contingent acquisition scenario leads to a higher donation level. These findings are consistent with Proposition 4.

**Proposition 5** Comparing the utilities under committed acquisition and contingent acquisition, we have the following results:

(a) when *c* < *c*_2_ or *c* > *c*_3_, the utility for supplier and profits for both retailer and the supply chain are equal under committed acquisition and contingent acquisition;

(b) when *c* ∈ (*c*_2_, *c*_1_), the profits for the retailer and the supply chain are higher under contingent acquisition, whereas the utility for the supplier is higher under committed acquisition;

(c) when *c* ∈ (*c*_1_, *c*_3_), the utility for supplier and profits for both retailer and the supply chain are always higher under committed acquisition.

Proposition 5 compares the results for both firms and the whole supply chain under the two strategies. The supplier receives higher utility by delaying his decision after the retailer acquisition, whereas the retailer’s profit depend on the situation. The different preferences of firms for the two strategies are influenced by the checks and balances of contradictory effects, which can be illustrated as follows.

First, from the retailer’s perspective, the positive side of contingent acquisition is its induction effect, which can induce the supplier to further invest in CM campaign. This situation subsequently allows the retailer to free-ride on this to derive more surplus from the end market. However, the downside of contingent acquisition is that it may also prevent the supplier’s incentive on investing in CM campaign and the retailer’s incentive of acquisition. The intuition is that under the contingent acquisition scenario, the retailer’s acquisition decision is entirely built upon the supplier’s donation level. When the acquisition cost is not that high and is affordable, the postponement of acquisition indeed incentives the supplier to invest more on CM charity. However, if the acquisition cost keeps increasing and becomes unaffordable, the supplier would cover the cost of a CM campaign, whose consequence is detrimental to the retailer.

Second, regarding the supplier, it is evident that committed acquisition should always dominate contingent acquisition. This is because in the latter acquisition strategy, the supplier has to occasionally overinvest in CM campaign, and the retailer possesses a lower incentive of acquisition. Both consequences are detrimental to the supplier’s profitability. Building upon the firm’s preference, it can be inferred that in a decentralized supply chain, the firms’ interests regarding the acquisition option can actually coincide with each other under certain conditions, despite that the conflicts in their other operational decisions still exist (e.g., pricing).

**Fig 3:**
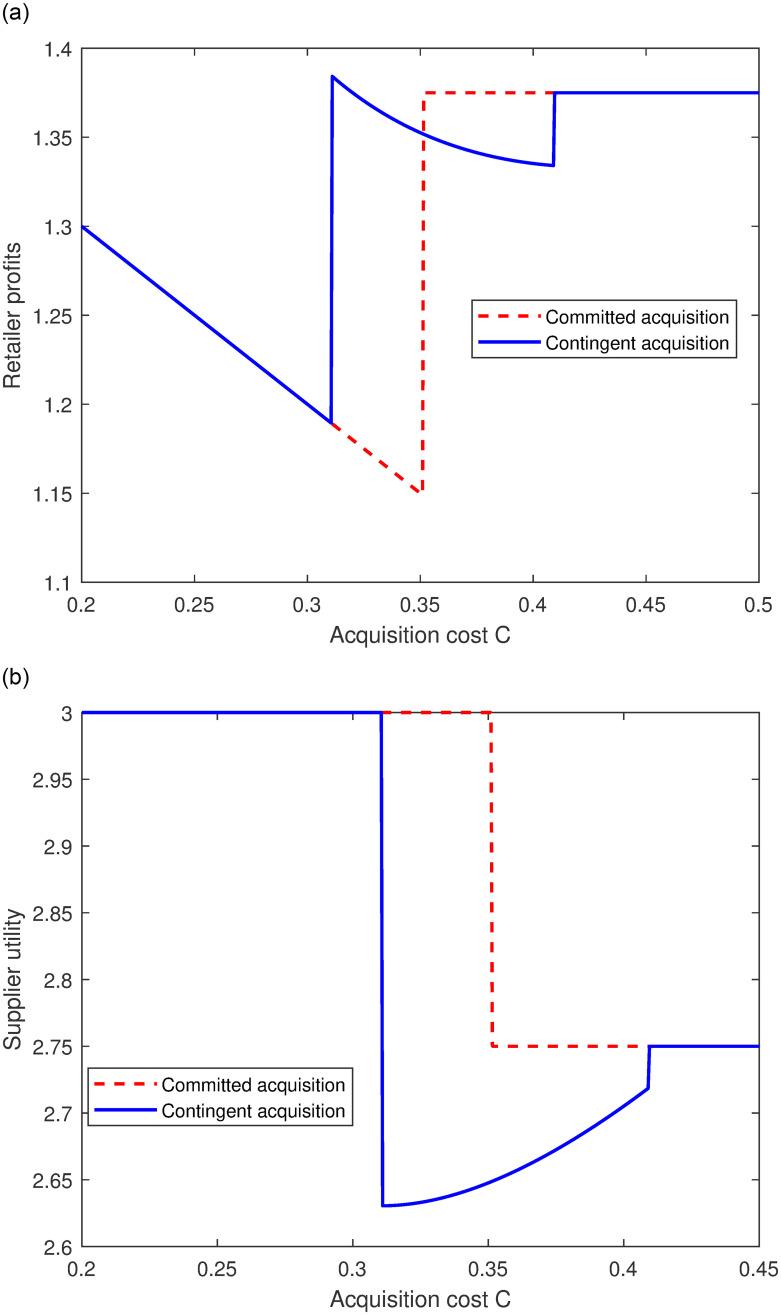
The supply chain members’ profit/utility. (a) Retailer’s profit; (b) Supplier’s utility.

Similar to Fig 2, we present the relationship between the acquisition cost and the supplier’s profit and retailer’s profit, as shown in Fig 3(a) and 3(b), respectively. From the retailer’s perspective, when the acquisition cost *c* is in a moderately low range (e.g., *c* ∈ (0.31, 0.35)), the positive side of contingent acquisition is induce the supplier to make a higher donation level. Because this effect makes the retailer to free-ride to derive more surplus. Thus, the contingent acquisition is the best choice for the retailer. In contrast, when *c* is in the moderately high range (e.g., *c* ∈ (0.35, 0.4)), the contingent acquisition may prevent the supplier’s incentive on investing in charity and the retailer’s incentive of acquisition. In this scenario, the retailer prefers committed acquisition to contingent acquisition. Regarding the supplier, the Fig 3 shows that committed acquisition should always dominate contingent acquisition. This is because in the committed acquisition strategy, supplier will only invest more when *c* is large (Proposition 4), and the retailer has a lower incentive of acquisition. Finally, both consequences are detrimental to the supplier’s utility.

**Fig 4:**
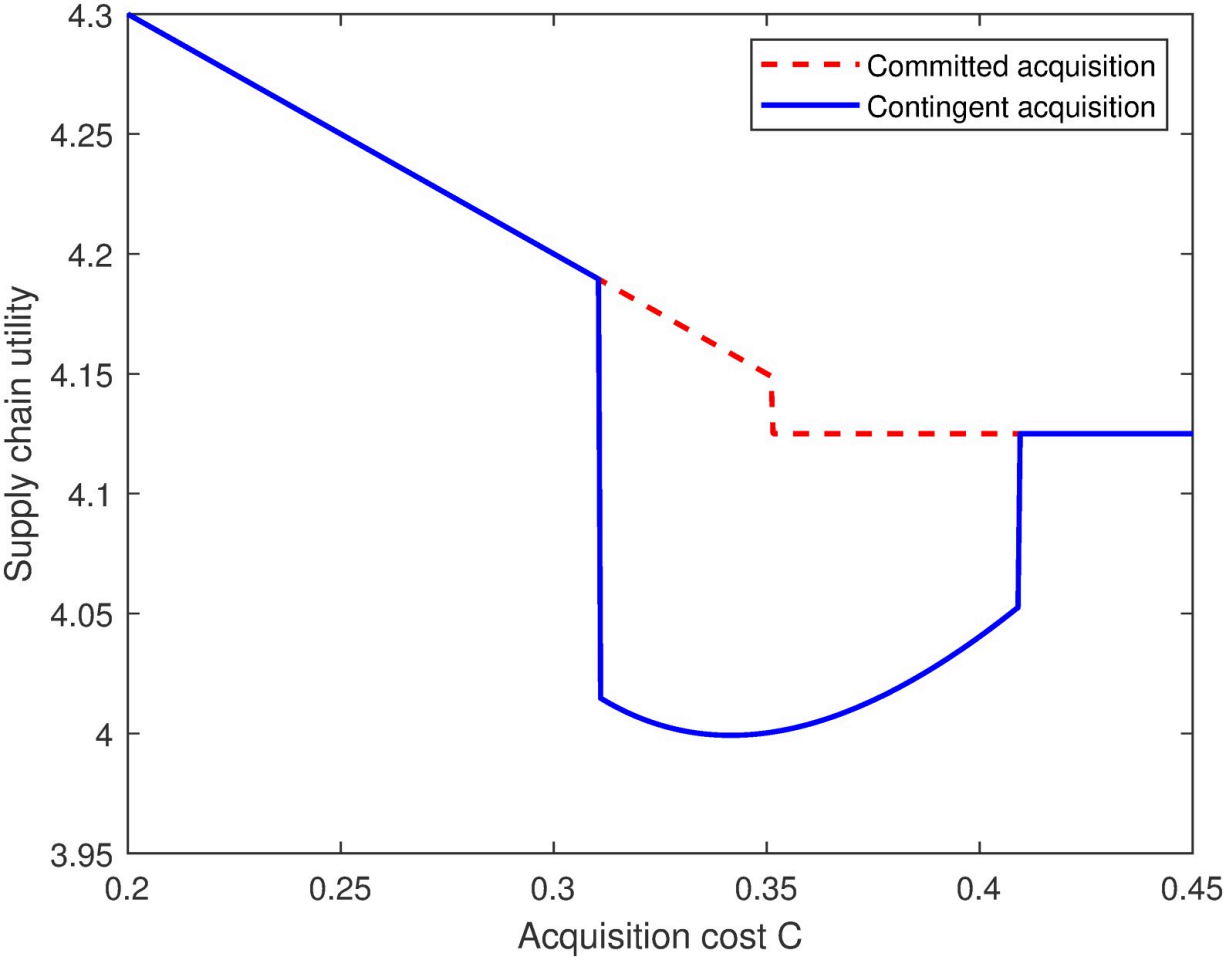
The supply chain utility.

Third, the supply chain’s preference over these two timing scenarios are the same as the supplier’s. Under the contingent strategy, despite damaging the profits of the supplier due to the higher donation cost, increased unit donation amount improves the performance of the whole supply chain. This is because the equilibrium donation level in a decentralized supply chain is lower than the optimal level in a centralized supply chain as a result of double marginalization, which can be alleviated by rasing the donation level under contingent acquisition. The graphical illustration is shown in Fig 4.

## 6 Discussion and conclusions

### 6.1 Discussion

As new generations (Millennials and Generation Z) enter the labor market and possess higher purchasing power in recent years, social issues become more prominent than ever before. Therefore, in recent years, the CM strategy has been used by several firms to enhance their socially responsible images, but how to effectively design CM is an urgent problem. While the previous research has identified “warm glow” as the primary driver of CM, which entice the consumers to buy the cause-linked products, the firms must be aware that the consumers are heterogeneous in their preferences for the CM. This also means neglecting heterogeneous of consumers’ in CM may lead the supply chain members to make decisions far from optimal. However, in real-life the supply chain often faces information asymmetry challenge. Thus, how to effectively identify consumer preference to CM is crucial for a firm engaged in CM campaign.

Based on this dilemma, we investigate a social responsibility supply chain wherein dual-purpose supplier sells his products through a for-profit retailer at a unit wholesale price via a retail channel. The supplier also engages in a CM campaign to charity. The consumers’ social preference is ex-ante unknown to both the supplier and retailer but can be gathered by the information acquisition behavior of the latter. We consider two information acquisition strategies: the committed and contingent strategies, which differ in terms of whether the information acquisition decision of the retailer is made prior to or after the supplier’s CM decision. By establishing game models, we derive the supplier’s equilibrium donation level, the retailer’s information acquisition behavior, and both firms’ pricing strategies under the two strategies and investigate how they affect the utilities of firms.

### 6.2 Conclusions

Through research, we obtain several interesting findings. First, compared with no-information acquisition, higher donation level is obtained with information acquisition under both strategies. In addition, we intriguingly find that, under the contingent strategy, the supplier may excessively invest in CM campaign to motivate the retailer to obtain consumes’ social preference information when the cost of information acquisition lies in a moderate level. Second, by analyzing the retailer’s information acquisition decision under both strategies, we demonstrate that under either acquisition strategy, the retailer always acquires information only when the acquisition cost is relatively small. Moreover, we uncover that, compared with the contingent one, the committed acquisition scheme boosts the retailer’s incentive to acquire information. Third, in equilibrium, the supplier always receives more utility under the committed strategy. However, for the retailer, he can achieve a better performance in either of the strategy, depending on the acquisition cost. Specifically, the retailer is indifferent between the two acquisition strategies when the acquisition cost is either sufficiently low or high; otherwise, when the cost of information acquisition is moderate, the retailer will shift er strategy from the contingent strategy to the committed strategy as the acquisition cost increases.

Our research suggests the following insights in managing socially responsible supply chains. Firstly, supply chain members participate in the CMs to contribute to their social responsibility imperatives and at the same time increase their sales. The CMs will succeed when they are well designed. Deciding the appropriate amount of donations is a key designated element of a CM that can lead supply chains to achieve their goals. Secondly, while the supply managers can count on warm glow effect to increase the demand of casue-linked products, the managers must be aware that consumers are heterogeneous in their preferences for social cause. Using appropriate acquisition strategies can help the supply chain managers to cope with the implied uncertainties. Thirdly, while the committed acquisition is more benefits for retailer, the contingent acquisition can induce the supplier set a higher donation amount and obtain a lower profit.

We point out a few caveats about our model and some directions for future research. First, a more complex consumer setting may be considered in future research. Our study merely considers two types of consumer social preferences (i.e., high and low), and future studies may consider additional types of consumers to generate highly complex yet interesting results. Second, we focus on a supply chain structure with a single dual-purpose supplier and single for-profit retailer. Future studies may consider examining a supply chain structure with multiple suppliers to establish more interesting interactions in both vertical and horizontal relationships. Third, with reference to [38], we use a theoretical model to investigate the interplay between the supplier’s CM decision and the retailer’s information acquisition, incorporating real-world data to support our model would be a better direction to improve its credibility.

## Supporting information

S1 Appendix (PDF)
