# Supplementary material for: Too Good to be Nice: The interplay between the Cause marketing and information acquisition
Source: PLoS One. 2024 May 23;19(5):e0299157. doi: 10.1371/journal.pone.0299157 (PMC11115330; doi:10.1371/journal.pone.0299157)
Supplement: S1 Appendix — (PDF) [file pone.0299157.s001.pdf]

Appendix

Too Good to be Nice: The interplay between the Cause marketing and information acquisition

No. PONE-D-23-37901

Proof of Proposition 1.

By comparing the equilibrium donation levels with acquisition and non-acquisition under the committed acquisition strategy, we can easy demonstrate that k^{N*} is always less than k^{A*}. $\square$

Proof of Proposition 2.

The retailer prefers information acquisition if and only if $\pi_r^A \geq \pi_r^N$, which is reduced to $c \leq c_1 = \frac{v[\sqrt{2+4\theta+4\theta^2}-1]}{16}$. Thus, the retailer prefers acquisition if $c \leq c_1$, and prefers non-acquisition otherwise. $\square$

Proof of Proposition 3

Given the retailer acquires information, her payoff is $\pi_r = \frac{[v+(1+\theta)k]^2}{16k} - c$ when $\beta = \beta_h$, or $\pi_r = \frac{(v+\theta k)^2}{16k} - c$ when $\beta = \beta_l$. While if the retailer chooses non-acquisition, her expected payoff is $\pi_r = \frac{[v+(1+\theta)k]^2}{32k}$. The retailer's optimal information acquisition decision arise from comparing her payoff under acquisition and non-acquisition. Thus, she prefers acquisition if and only if $c \leq \frac{(v+\theta k)^2}{32k}$. According to the retailer's optimal decision on information acquisition strategy, we then move to the supplier's optimal donation decision. In particular, we consider the following two cases:

(a) If the retailer opts to obtain information about consumers' preferences information for social cause, then the upstream supplier solves the following optimization problem:

$$\max \pi_s^A(k) = \underbrace{\frac{1}{2}\frac{[v+(1+\theta)k]^2}{8k}}_{\beta=\beta_h} + \underbrace{\frac{1}{2}\frac{(v+\theta k)^2}{8k}}_{\beta=\beta_l},$$
$$s.t., \quad c \leq \frac{(v+\theta k)^2}{32k}.$$

We can get the optimal solution to the optimization problem as follows: If $c < \frac{v(2\theta+\sqrt{2+4\theta+4\theta^2})^2}{64\sqrt{2+4\theta+4\theta^2}}$, then $k^* = \frac{2v}{\sqrt{2+4\theta+4\theta^2}}$; otherwise, $k^* = \frac{16c-\theta v-4\sqrt{16c^2-2c\theta v}}{\theta^2}$. Therefore, the supplier's utility is $\pi_s^A = \frac{v[1+2\theta+\sqrt{2+4\theta+4\theta^2}]}{8}$ if $c < \frac{v(2\theta+\sqrt{2+4\theta+4\theta^2})^2}{64\sqrt{2+4\theta+4\theta^2}}$; otherwise

$$\pi_s^A = \frac{8\sqrt{16c^2-2vc\theta}[\theta v(1+\theta)-16(2\theta^2+2\theta+1)c]+512c^2(2\theta^2+2\theta+1)+32v\theta c(2\theta^2+3\theta+2)+\theta^2v^2}{16\theta^2(16c-\theta v-4\sqrt{16c^2-2c\theta v})}.$$

(b) If the retailer gives up to obtain information about consumers' social preference information, then the upstream supplier solves the following optimization problem:

$$\max \pi_s^N(k) = \frac{[v+(1+\theta)k]^2}{16k},$$
$$s.t., \quad c > \frac{(v+\theta k)^2}{32k}.$$

The optimal solutions are $k^* = \frac{v}{1+\theta}$ if $c > \frac{v(1+2\theta)^2}{32(1+\theta)}$ and $k^* = \frac{16c-\theta v-4\sqrt{16c^2-2c\theta v}}{\theta^2}$ otherwise. Thus, the supplier's utility is $\pi_s^N = \frac{v(1+\theta)}{4}$ if $c > \frac{v(1+2\theta)^2}{32(1+\theta)}$; otherwise $\pi_s^N = \frac{\{4(1+\theta)(\sqrt{16c^2-2c\theta v}-4c)+\theta v\}^2}{16\theta^2[16c-\theta v-4\sqrt{16c^2-2c\theta v}]}$.

According to the supplier's and retailer's equilibrium profits with acquisition and non-acquisition cases, the following inequality holds:

$$\begin{cases} \frac{v[1+2\theta+\sqrt{2+4\theta+4\theta^2}]}{8} \geq \frac{8\sqrt{16c^2-2vc\theta}[\theta v(1+\theta)-16(2\theta^2+2\theta+1)c]+512c^2(2\theta^2+2\theta+1)+32v\theta c(2\theta^2+3\theta+2)+\theta^2 v^2}{16\theta^2(16c-\theta v-4\sqrt{16c^2-2c\theta v})}, \\ \frac{v[1+2\theta+\sqrt{2+4\theta+4\theta^2}]}{8} \geq \frac{v(1+\theta)}{4}, \\ \frac{v(1+\theta)}{4} \geq \frac{\{4(1+\theta)(\sqrt{16c^2-2c\theta v}-4c)+\theta v\}^2}{16\theta^2[16c-\theta v-4\sqrt{16c^2-2c\theta v}]}. \end{cases}$$

Thus, (1) when the acquisition cost $c \leq c_2 = \frac{v(2\theta+\sqrt{2+4\theta+4\theta^2})^2}{64\sqrt{2+4\theta+4\theta^2}}$, the retailer opts to obtain consumers' social preference information; (2) when the acquisition cost $c > c_2$, acquisition dominates non-acquisition if $\pi_s^A \geq \pi_s^N$. After some simplifications, we obtain $c_2 < c \leq c_3 = \frac{v(8\theta^3+12\theta^2+6\theta+3+\sqrt{48\theta^3+128\theta^3+120\theta^2+48\theta+7})}{64(1+2\theta+2\theta^2)}$. (3) when $c \geq c_3$, the upstream supplier just induces the retailer not to obtain consumers' social preference information. □
